# *mRNA* microarray data of FACS purified bovine small and large luteal cells

**DOI:** 10.1016/j.dib.2018.05.029

**Published:** 2018-05-24

**Authors:** Vijay S. Baddela, Arpna Sharma, Dirk Koczan, Torsten Viergutz, Andreas Vernunft, Jens Vanselow

**Affiliations:** a Reproductive Biology, Leibniz Institute for Farm Animal Biology (FBN), 18196 Dummerstorf, Germany; b Institute for Immunology, University of Rostock, 18055 Rostock, Germany

## Abstract

Transcriptome profiling of FACS purified small luteal cells (SLC) and large luteal cells (LLC), isolated from mid-cycle corpora lutea (day 10–11), was performed using Affymetrix GeneChip Bovine Gene 1.0 ST Arrays. Gene expression was recorded using an Affymetrix gene chip scanner 3000. The corresponding expression array intensity (.CEL) files were deposited in the Gene Expression Omnibus (GSE106641). The subsequent expression analyses of CEL files were carried out for identifying important transcripts and their functions associated with SLC and LLC. These data have been comprehensively evaluated and interpreted in the companion article, “Global gene expression analysis indicates that small luteal cells are involved in extracellular matrix modulation and immune cell recruitment in the bovine corpus luteum” [1].

**Specifications Table**

**Table:** 

Subject area	Transcriptomics
More specific subject area	Bovine reproduction
Type of data	Table, figure
How data was acquired	RNA microarray analysis using Affymetrix GeneChip Bovine Gene 1.0 ST Arrays
Data format	Normalized, analyzed and filtered
Experimental factors	FACS purified small and large luteal cells
Experimental features	A comparative transcriptome analysis of small and large luteal cells isolated from specifically staged bovine mature corpora lutea by fluorescence-activated cell sorting (FACS)
Data source location	Leibniz Institute for Farm Animal Biology (FBN,) 18196 Dummerstorf, Germany
Data accessibility	The analyzed data are present in the supplementary tables. Raw microarray datasets were deposited in NCBI GEO and can be accessed at https://www.ncbi.nlm.nih.gov/geo/query/acc.cgi?acc=GSE106641

**Value of the data**

• There are no previously published microarray data derived from small luteal cells (SLC) and large luteal cells (LLC) that were isolated from mid-cycle mature corpora lutea.
• The present transcriptome data can be compared with previously published granulosa and theca cell transcriptomes [2], [3], [4] to understand the molecular changes behind the folliculo-luteal transformation.
• The identified differentially expressed genes can inform future physiological research on luteal cell function and dysfunction.

## 1 Data

**Fig. 1:**
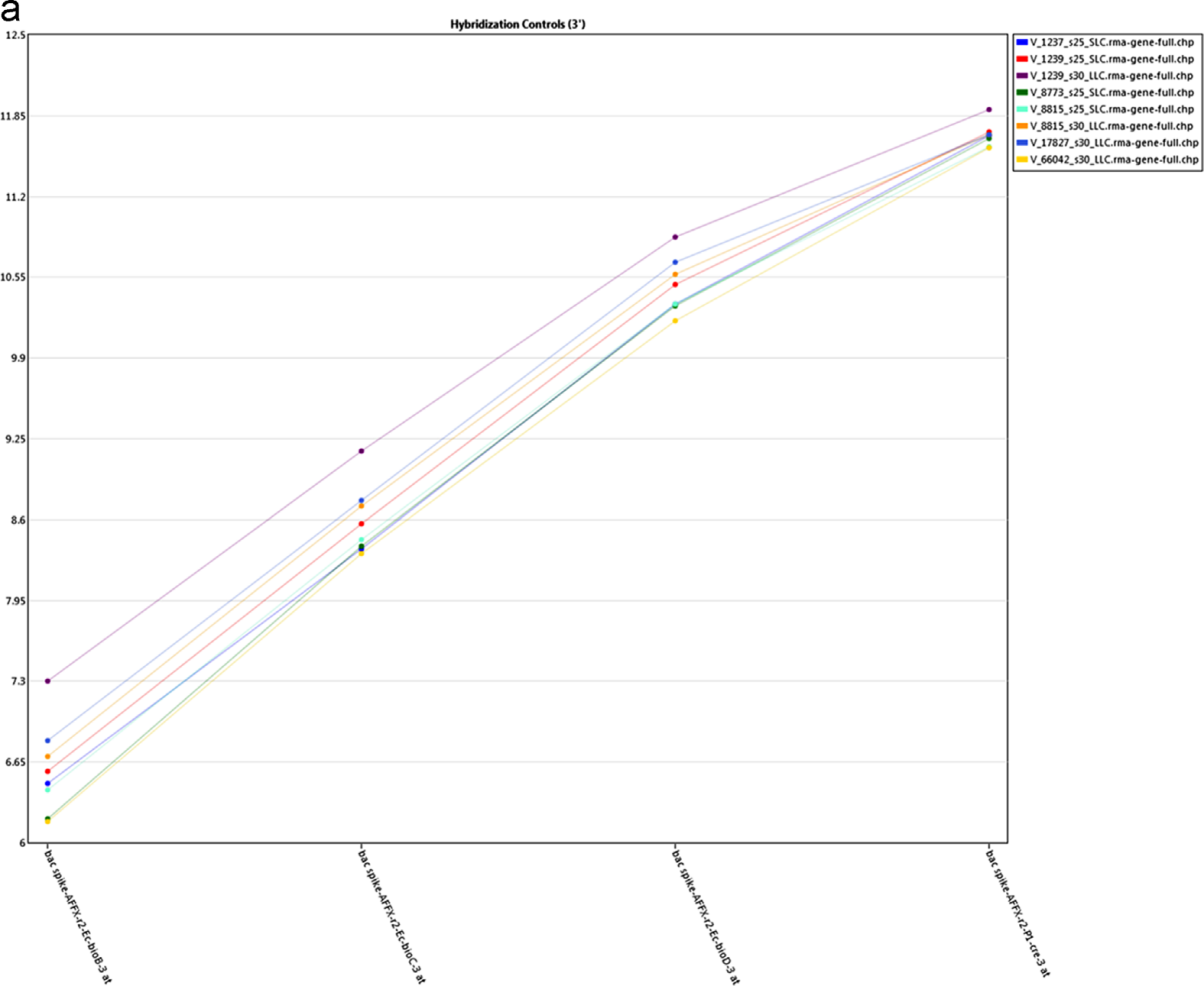

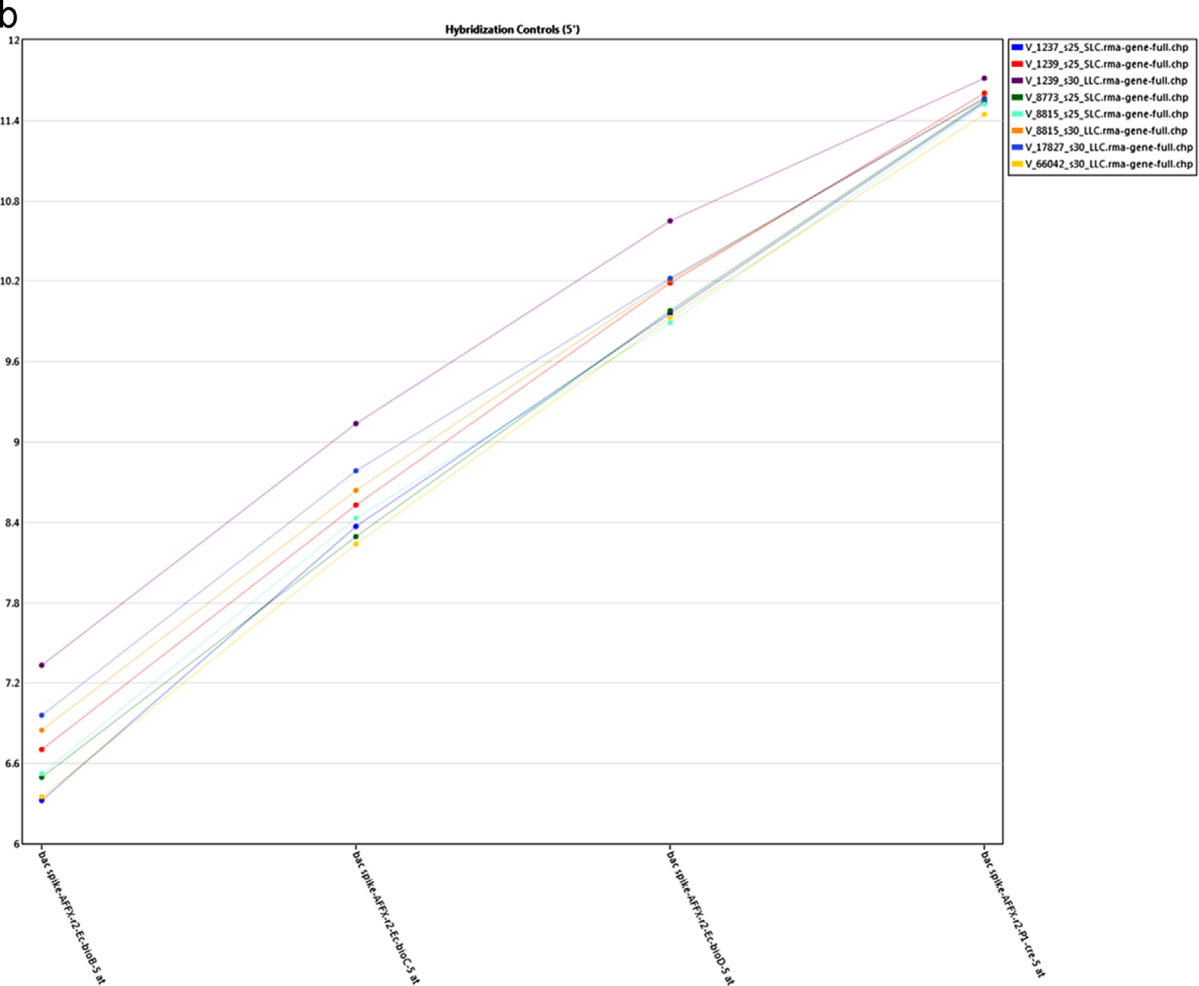

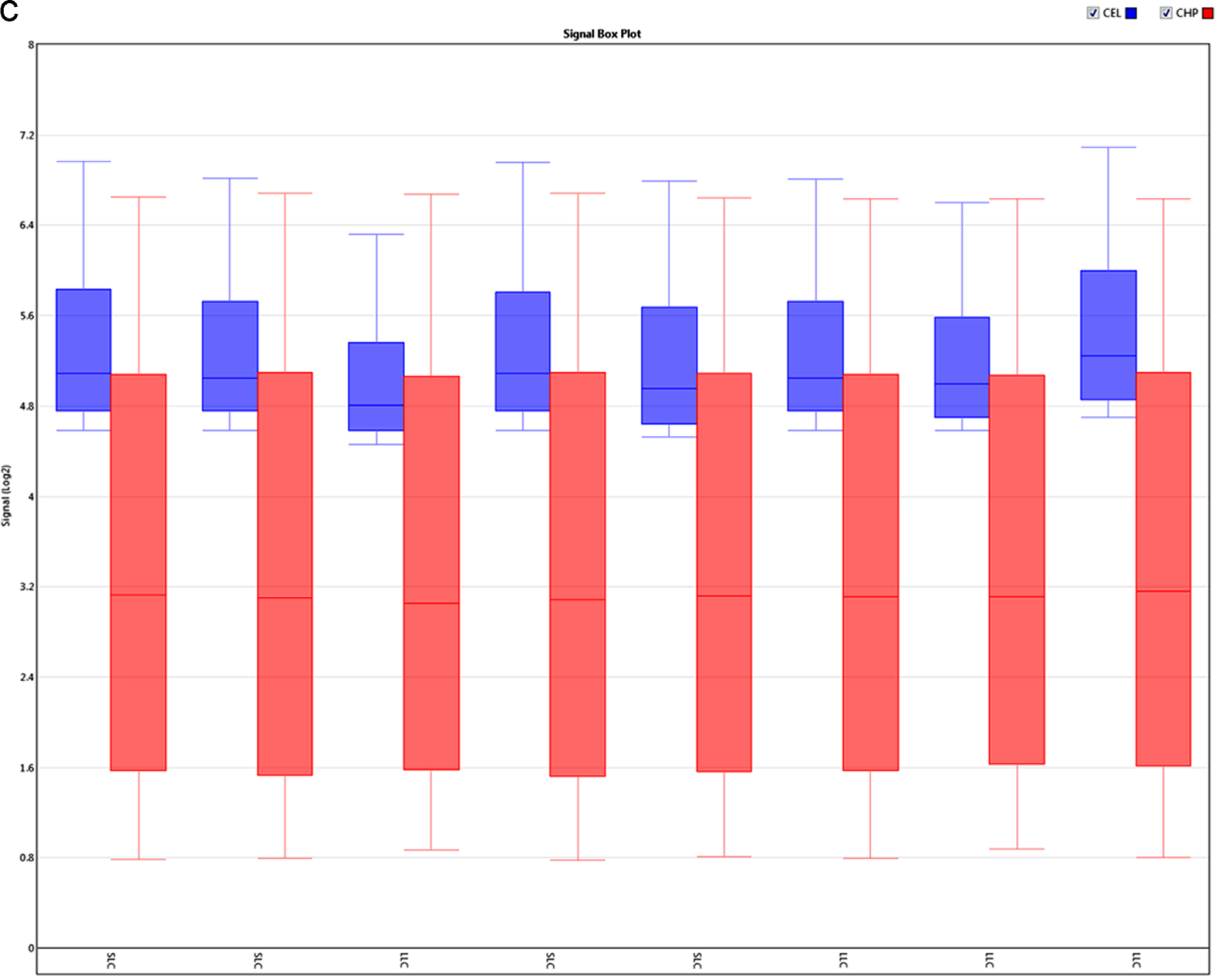
a: Signal data from 3′ hybridization controls from all samples. b: Signal data from 5′ hybridization controls from all samples. c: Signal box plots of array files before (CEL) and after (CHP) normalization.

**Fig. 2:**
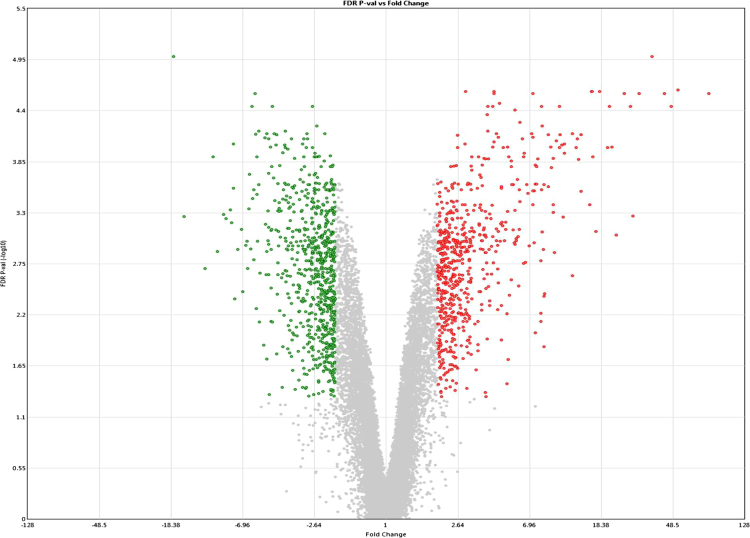
Volcano plot visualization of differentially expressed genes.

The present microarray experiment was performed using the Affymetrix GeneChip Bovine Gene 1.0 ST Arrays. The generated raw data files were deposited at NCBI GEO repository series GSE106641 (https://www.ncbi.nlm.nih.gov/geo/query/acc.cgi?acc=GSE106641). Analysis of raw expression array files was carried out using the Transcriptome Analysis Console 4.0 software package. Evaluation of signal data from 3′ and 5′ hybridization controls (Fig. 1a and b), and normalized signal box plots in the “Sample QC View” tab of TAC 4.0 software (Fig. 1c) revealed that sample processing and data acquisition steps have passed in the quality control (QC) checkup (Supplementary word file 1). The subsequent evaluation of gene expression data identified expression values for 20,423 annotated gene clusters (Table 1), which were further filtered (|FC| > 2; p < 0.05 and FDR q < 0.05) for the identification of differentially expressed genes (Fig. 2). This resulted in spotting of 1276 DEG between small and large luteal cells (Table 2). To visualize the expression of individual DEG an interactive heatmap was generated using an R package (shinyHeatmaply), which is provided as a supplementary html file. The IPA analysis resulted in the identification of 374 (Table 3) and 500 (Table 4) enriched canonical pathways and biological functions, respectively, for DEG. Further, a protein protein interaction (PPI) network between DEG was also identified using networkAnalyst tool. The hub genes were recognized based on “degree” and “betweeness” of interaction with other proteins in the PPI network (Tables 5 and 6).

## 2 Experimental design, materials and methods

Like in many species, CL of cows contain small and large luteal cells, which are derived from follicular granulosa and theca cells [5], [6]. The present study was designed to characterize the transcriptomes of SLC (n = 4) and LLC (n = 4) isolated from mid-cycle corpora lutea. Cows were synchronized by injecting 0.5 mg cloprostenol (PGF Veyx forte®). On Day 10 or 11 of the successive estrus cycle, mature CL was collected from the estrus synchronized animals via colpotomy. CL were immediately transferred into ice cold PBS and taken to the laboratory for washing and disaggregation. Next, CL were finely sliced and incubated in 1× Hank׳s solution containing 0.1% collagenase for 45 min at 37 °C with continued stirring. The dissociated cells were filtered through a stainless screen. The cells in the filtrate were transferred into a new collection tube and centrifuged to pellet the cells. The undigested tissue chunks present on the screen were again disaggregated until CL is completely dissociated. The dissociated luteal cells were subjected to a discontinues percoll gradient centrifugation to collect the enriched luteal cell fractions, containing small and large luteal cells. These mixed luteal cells were stained with Hoechst 33342 dye and sorted by FACS to collect the small and large luteal cell fractions, separately. The average diameter of isolated LLC and SLC were 31 ± 3 μm and 15 ± 2, respectively. RNA was isolated from the luteal cells using the RNeasy mini kit (Qiagen, Hilden, Germany) and subjected to transcriptome analysis using the GeneChip™ Bovine Gene 1.0 ST Array (Affymetrix®, Inc., Santa Clara, CA, USA). After analyzing the RNA quality using a bio analyzer instrument (Agilent technologies, ST Clara, CA, USA), RNA samples were amplified and labelled with GeneChip 3’amplification and one-cycle target labelling reagents as recommended by Affymetrix. Next, all the samples were hybridized overnight and gene expression signals were acquired using Affymetrix Gene Chip Scanner 3000. The acquired data were processed and analyzed using the Transcriptome Analysis Console 4.0 (TAC 4.0. affymetrix). DEG were identified using filter parameters, fold difference >|2|, ANOVA p < 0.05, and FDR q < 0.05. The enriched canonical pathways and biological functions were identified using the Ingenuity pathway analysis tool (IPA, Qiagen, Hilden). The “NetworkAnalyst” tool, accessible at www.Networkanalyst.ca, was used to construct a PPI network that was subsequently used for the identification of hub genes based on “degree” or “betweenness” of interaction with other proteins.
