# Supplementary material for: mRNA microarray data of FACS purified bovine small and large luteal cells
Source: Data Brief. 2018 May 24;19:737–42. doi: 10.1016/j.dib.2018.05.029 (PMC5997839; doi:10.1016/j.dib.2018.05.029)
Supplement: Supplementary file 2 — Supplementary material [file mmc2.docx]

**Quality parameters of microarray data:**

**Hybridization controls:**

Four external hybridization controls (AFFX-r2-Ec-BioB, AFFX-r2-Ec-BioC, AFFX-r2-Ec-BioD, AFFX-r2-P1-Cre) were added to the samples to evaluate the hybridization efficiency of microarray experiment. These controls should display an increasing signal values (AFFX-r2-Ec-BioB<AFFX-r2-Ec-BioC<AFFX-r2-Ec-BioD<AFFX-r2-P1-Cre), reflecting their relative concentrations, in order to pass the quality parameters. The TAC4.0 software will automatically read the hybridization control values and show the result as “Pass” or “Out” for all samples. All the samples in the current microarray experiment have been shown as “pass” with respect to hybridization controls.

**Signal box plots:**

Signals box plots can be generated to compare the array files before (CEL) and after (CHP) normalization of microarray experiment. A general rule to examining the signal box plots is to look for the individual arrays that are different from one another. The normalized probe set intensities are expected to be more similar compared to un-normalized intensities of different array files. Drawing the box plots before and after the normalization step allows checking the normalization step of microarray data. The current microarray data showed similar intensity values for the normalized data files (CHP), indicating no potential problems concerning the processing of microarray data.
