# Supplementary material for: mRNA microarray data of FACS purified bovine small and large luteal cells
Source: Data Brief. 2018 May 24;19:737–42. doi: 10.1016/j.dib.2018.05.029 (PMC5997839; doi:10.1016/j.dib.2018.05.029)
Supplement: Supplementary file 3 — Supplementary material [file mmc3.zip › mmc3.html]

| Parameter | Value |
| --- | --- |
| main | NULL |
| xlab | NULL |
| ylab | NULL |
| row\_text\_angle | 0 |
| column\_text\_angle | 45 |
| dendrogram | both |
| branches\_lwd | 0.6 |
| seriate | OLO |
| colors | viridis(256) |
| distfun\_row | euclidean |
| hclustfun\_row | complete |
| distfun\_col | euclidean |
| hclustfun\_col | complete |
| k\_col | 2 |
| k\_row | 2 |
| limits | NULL |

*This heatmap visualization was created using shinyHeatmaply 2018-02-28 16:23:27*
